# Antamanide Analogs as Potential Inhibitors of Tyrosinase

**DOI:** 10.3390/ijms23116240

**Published:** 2022-06-02

**Authors:** Claudia Honisch, Matteo Gazziero, Roberto Dallocchio, Alessandro Dessì, Davide Fabbri, Maria Antonietta Dettori, Giovanna Delogu, Paolo Ruzza

**Affiliations:** 1 Institute of Biomolecular Chemistry of CNR, Padova Unit, Via F. Marzolo, 1, 35131 Padova, Italy; c.honisch@icb.cnr.it (C.H.); matteo.gazziero@gmail.com (M.G.); 2 Institute of Biomolecular Chemistry of CNR, Sassari Unit, Traversa La Crucca, 3, 07100 Sassari, Italy; roberto.dallocchio@icb.cnr.it (R.D.); alessandro.dessi@cnr.it (A.D.); davidegaetano.fabbri@cnr.it (D.F.); mariaantonietta.dettori@cnr.it (M.A.D.); giovanna.delogu@icb.cnr (G.D.)

**Keywords:** tyrosinase inhibition, antamanide, bioactive peptides, UV-spectroscopy, computational docking

## Abstract

The tyrosinase enzyme, which catalyzes the hydroxylation of monophenols and the oxidation of *o*-diphenols, is typically involved in the synthesis of the dark product melanin starting from the amino acid tyrosine. Contributing to the browning of plant and fruit tissues and to the hyperpigmentation of the skin, leading to melasma or age spots, the research of possible tyrosinase inhibitors has attracted much interest in agri-food, cosmetic, and medicinal industries. In this study, we analyzed the capability of antamanide, a mushroom bioactive cyclic decapeptide, and some of its glycine derivatives, compared to that of pseudostellarin A, a known tyrosinase inhibitor, to hinder tyrosinase activity by using a spectrophotometric method. Additionally, computational docking studies were performed in order to elucidate the interactions occurring with the tyrosinase catalytic site. Our results show that antamanide did not exert any inhibitory activity. On the contrary, the three glycine derivatives **AG9**, **AG6**, and **AOG9**, which differ from each other by the position of a glycine that substitutes phenylalanine in the parent molecule, improving water solubility and flexibility, showed tyrosinase inhibition by spectrophotometric assays. Analytical data were confirmed by computational studies.

## 1. Introduction

Mushrooms represent a significant source of natural bioactive peptides [1]. Indeed, in mushrooms not only bioactive peptides derived from protein fragmentation are present, but also small peptides, linear or cyclic, synthesized by mushrooms themselves [2].

In the past years, we evaluated the chemical and biological activities of the cyclic decapeptide antamanide, firstly isolated from the lipophilic fraction of an extract of *Amanite phalloides* by the T. Wieland’s group in 1962 [3]. A characteristic of this peptide is its ability to form complexes of high stability with metal ions, including Na^+^, K^+^, Ca^2+^, Tl^3+^, and others [4,5,6,7,8]. A close correlation has been found between the ion binding properties and the selectivity for Na^+^ over K^+^ with its antitoxic activity against phallotoxins and the prevention of their accumulation in the liver cells [9]. Successive studies showed that antamanide possesses other biological activities, including an immunosuppressive activity comparable to that of cyclosporine A [10,11]. Moreover, antamanide treatment is also effective in limiting lung and heart edema [12], as well as preventing the development of neoplastic cells in mice models of leukemia [13]. Successively, the activity of antamanide and some linear and cyclic derivatives has also been tested on metastatic melanoma cell line, finding that antamanide caused a significant reduction of cell proliferation after 24 h of treatment, while its glycine derivatives were initially inactive and showed a cytostatic activity only after 48 h from the treatment [14].

**Scheme 1:**
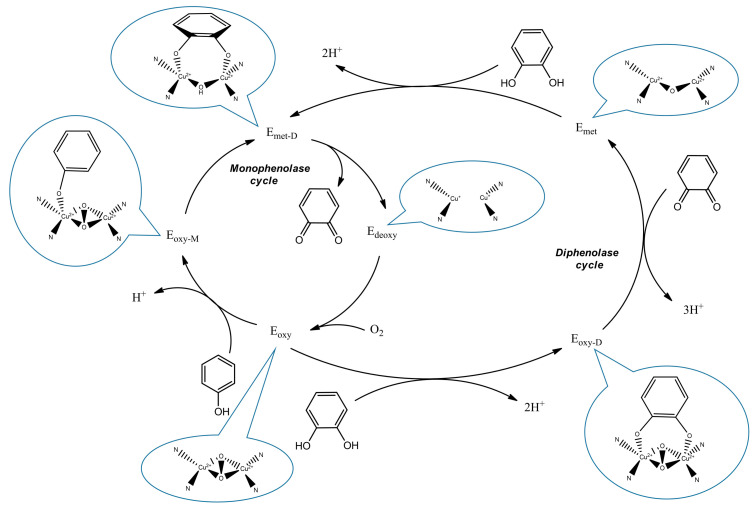
Representation of tyrosinase states during monophenolase and diphenolase activity and catalytic site conformation in the blue balloons.

Melanoma is an aggressive tumor that over-expresses tyrosinase [15], a widely distributed enzyme throughout the phylogenetic scale from bacteria to mammals. This copper-containing enzyme catalyzes two distinct reactions: the hydroxylation of monophenols and the oxidations of *o*-diphenols to *o*-quinones. Although the typical substrate of tyrosinase is represented by the amino acid *L*-tyrosine (*L*-Tyr), tyrosinase activities appear to have broad substrate specificities. In the hydroxylation reaction, also known as monophenolase or creolase activity, the enzyme passes through four different states (E_deoxy_, E_oxy_, E_oxy-M_, and E_met-D_), while in the successive oxidation of *o*-diphenols, known as diphenolase or cathecolase activity, five enzyme states (E_deoxy_, E_oxy_, E_oxy-D_, E_met_, and E_met-D_) are involved (Scheme 1) [16].

The *o*-quinones are generally reactive and can sustain 1,4-addition to the benzene ring to provide, after several steps, eumelanin and pheomelanin, the prototypes of melanin [17]. In fungi and vertebrates, tyrosinase catalyzes the initial step in the formation of the pigment melanin from tyrosine. In plants, the physiological substrates are a variety of phenolics that are oxidized in the browning pathway observed when tissues are injured. Indeed, the first biochemical investigations on this enzyme were carried out in 1895 on the mushroom *Russula nigricans*, whose cut flesh turns red and then black on exposure to air [18].

Considering the important role that tyrosinase plays in the processing of fruit and vegetables and during the storage of processed foods, as well as in the hyperpigmentation of the skin with melasma and age spots, its inhibition is attractive in cosmetic, medicinal, and food industries. For this purpose, many natural and synthetic inhibitors have been developed [19] with the aim to obtain new safe and efficient anti-tyrosinase agents for the prevention of browning in plant-derived foods, seafood, and hyperpigmentation treatments.

**Figure 1:**
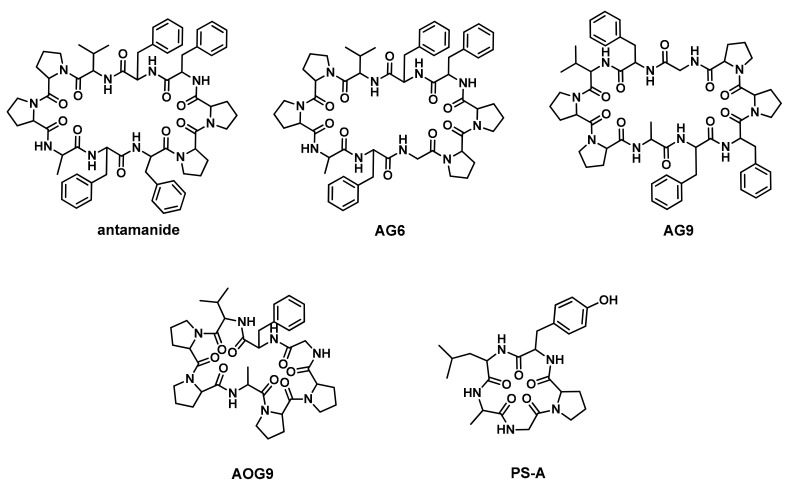
Chemical structure of the investigated cyclic peptides.

In this study, we analyzed the capability of the bioactive peptide antamanide and some of its Gly-derivatives (Table 1 and Figure 1) to inhibit tyrosinase activity. The substitution of Phe residue with Gly improves the water solubility of antamanide as well as the flexibility. The activity of antamanide derivatives was compared to that of pseudostellarin A (**PS-A**), a cyclic peptide isolated from *Pseudostellaria heterophylla* able to inhibit the tyrosinase activity [20]. A computational approach as molecular docking was used to understand at a molecular level the interactions occurring between the enzyme catalytic site and peptide inhibitors.

## 2. Results

### 2.1. Peptide Design and Synthesis

Antamanide is a cyclic decapeptide isolated from the lipophilic fraction of an extract of the green toadstool *Amanite phalloides*, able to form complexes of high stability with different metal ions [4,5,6,7,8,9]. In order to improve the hydrophilicity of this peptide, the Phe residue at either the six or nine positions was replaced with a Gly residue. Previous studies showed that the substitution of Gly for Phe at these positions did not induce loss of the characteristic ion binding properties, maintaining the ions selectivity determined for antamanide (Ca > Na >> K) [21], while their cytotoxic activity on B16F10 metastatic cells is reduced compared to the parent peptide antamanide. Indeed, only the **AG9** peptide showed a comparable activity after 48 h of treatment, while both **AG6** and **AOG9** resulted in being non-cytotoxic [14].

Pseudostellarin A is a cyclic pentapeptide isolated from the roots of *Pseudostellaria heterophylla* [22] characterized by a tyrosinase inhibitory activity and, in this study, was used as a reference standard to compare the inhibitory activity of the antamanide analogs.

The linear peptides were synthesized by manual solid phase peptide synthesis starting from a preloaded Fmoc-Ala-Wang resin using the Fmoc/HBTU chemistry [23]. To prevent the formation of byproducts due to deletion reactions either in the Pro-Pro or in the Xaa-Pro sequences, double coupling of either Fmoc-Pro-OH or Fmoc-Xaa-OH residue was performed using HATU as coupling agent. After assembly of the peptide, it was detached from the resin by treatment with TFA, and the crude peptide was cyclized in diluted DMF solution (1 mM) by addition of DPPA (diphenylphosphoryl azide, 3 eq) as a coupling reagent in the presence of a low soluble inorganic base (K_2_HPO_4_, 5 eq). After RP-HPLC purification, the peptides were obtained in good yield with a purity of 95%.

### 2.2. Tyrosinase Inhibition

**Figure 2:**
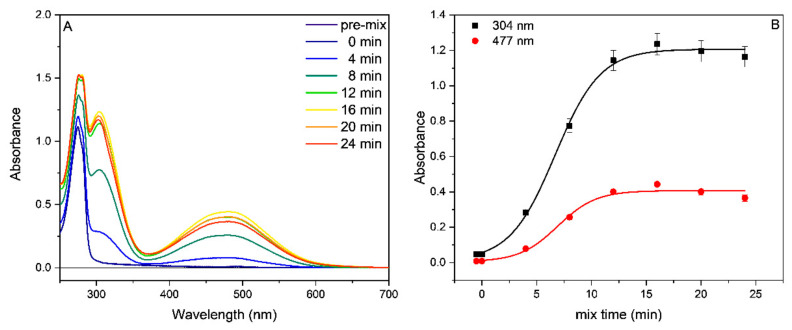
(**A**) UV-Vis spectra of *L*-Tyr (2 mM) in 20 mM phosphate buffer, pH 7.4, pre-mix and after mix with the tyrosinase solution (0.12 mg/mL) at different time of incubation (indicated). (**B**) Time-course of tyrosine oxidation by the tyrosinase/O_2_ oxidizing system. Data are the average of three replicates and the error is less than 5%.

**Scheme 2:**
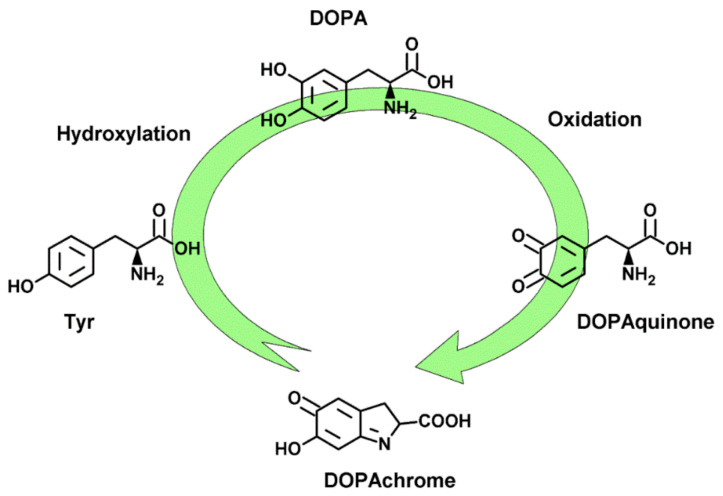
Products of tyrosine hydroxylation and oxidation by tyrosinase.

The capability of peptides to inhibit the activity of tyrosinase has been evaluated by a UV-Vis spectroscopic method monitoring the changes in the UV-Vis spectrum of its natural substrate, *L*-Tyr. As shown in Figure 2A, the near-UV spectrum of tyrosine in a buffer solution before the addition of enzyme is characterized by the presence of a band at about 275 nm due to the L*_b_* transition in the Platt’s notation [24] of the phenolic moiety of the tyrosine residue. The addition of enzyme firstly hydroxylated the *L*-Tyr residue to dihydroxyphenylalanine (DOPA) and successively oxidized DOPA to dopaquinone, which is then converted to dopachrome (Scheme 2). Both DOPA and dopaquinone appearance can be detected by the change in the UV-Vis spectrum. The hydroxylation of *L*-Tyr to DOPA is characterized by an increase in the intensity of the band at 275 nm and the appearance of an additional band at about 304 nm, while the oxidation to dopaquinone is made detectable by the appearance of a band at 470–490 nm. The time-course of the tyrosine oxidation (Figure 2B), obtained by monitoring the absorbance either at 477 or at 304 nm, showed the characteristic lag-phase of reaction catalyzed by the tyrosinase enzyme. This phase is related to the monophenolase activity of the enzyme and essentially is due to the low amount of the *oxy* form of the tyrosinase enzyme in the commercially available preparation. Although other various factors may play an important role, including substrate and enzyme concentration, enzyme source, pH of the medium, and presence of hydrogen donors such as DOPA or other catechols, also the presence of transition metal ions can influence the presence and the length of this lag-phase.

Millimolar peptide stock solutions were prepared in DMSO and then diluted to μM concentration by buffer in UV cells. The absence of any scattering signals in the UV spectra indicated that peptides and tyrosinase did not aggregate or precipitate [25]. The capability of peptides to inhibit the tyrosinase activity was evaluated at two different peptide concentrations, 2 and 0.2 µM. At both these concentrations, the parent peptide antamanide did not inhibit the tyrosinase activity, and the time courses are superimposable to that obtained for tyrosine alone (Figure S1 in Supplementary Materials). The absence of effects on tyrosinase activity was conserved also increasing the antamanide concentration to 20 µM (data not shown).

**Figure 3:**
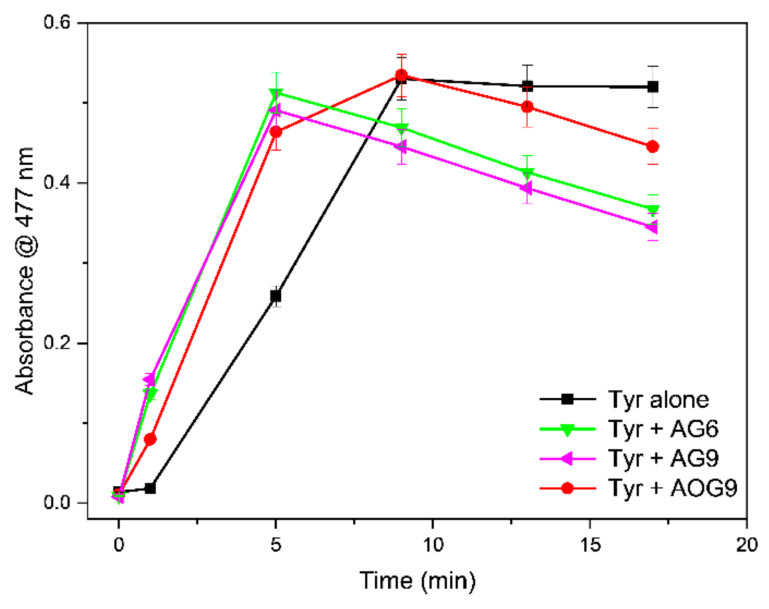
Influence of antamanide derivatives (0.2 µM) on the time-course of tyrosine oxidation by the tyrosinase/O_2_ oxidizing system. Data are the average of three replicates and the error is less than 5%.

**Figure 4:**
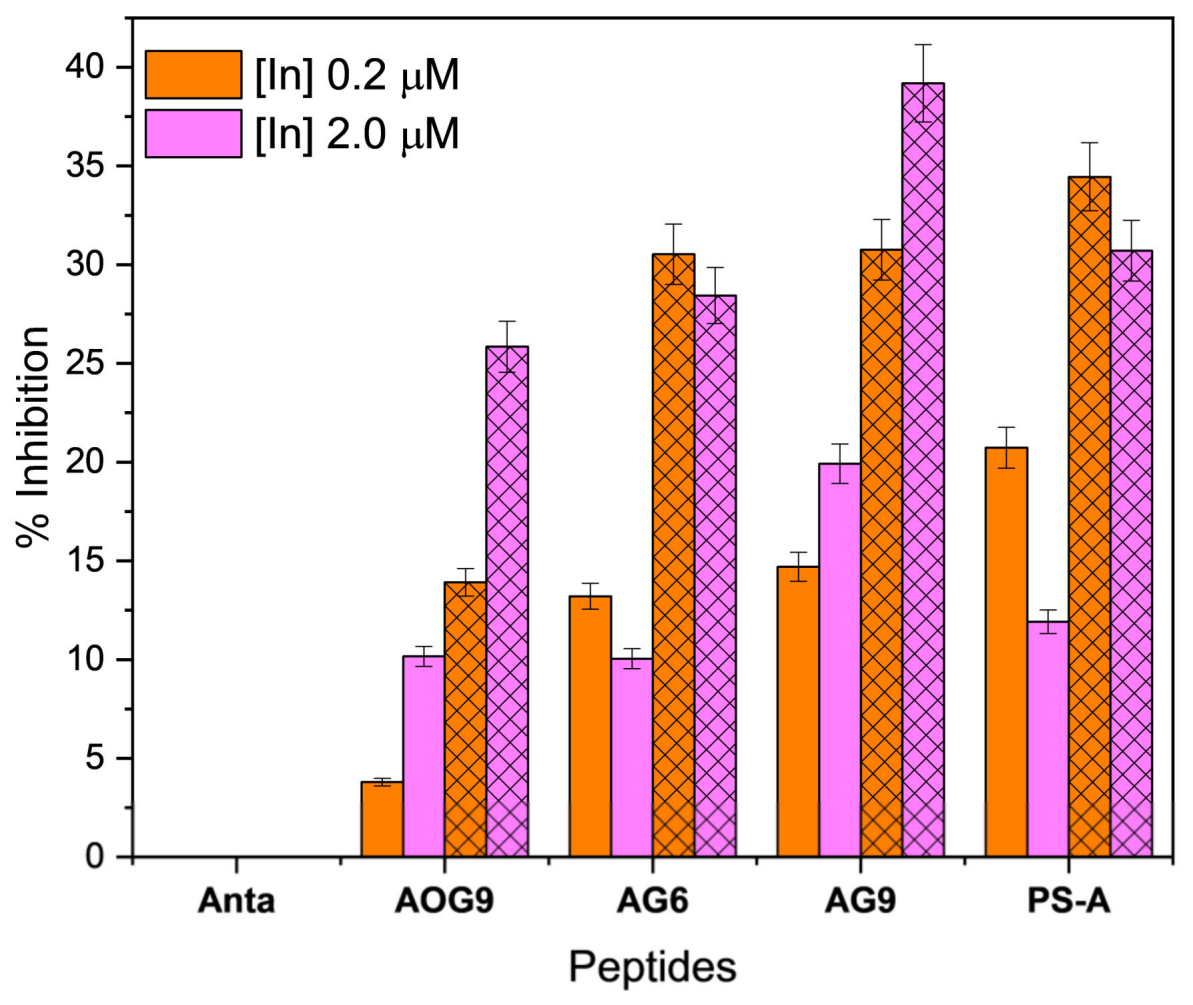
Percentage of tyrosinase inhibition determined by UV-VIS spectroscopy at 303 (color) and 477 nm (color plus grid) at different concentration of inhibitors (indicated).

In the presence of antamanide derivatives as well as of **PS-A**, a decrease in the tyrosinase activity has been detected, and the lag time phase observed for the Tyr substrate alone disappeared, while the band at 477 nm due to the dopaquinone and dopachrome reached a maximum and then decreased in time-dependent mode (Figure 3 and Figure 4).

In these conditions, among the investigated cyclopeptides, **AG9** showed higher tyrosinase inhibitory activity, whereas **AOG9** resulted in being the less active one (Figure 3 and Figure 4). The inhibitory efficiency of antamanide derivatives, calculated at 303 and 477 nm, showed the presence of a selective activity towards the two reactions catalyzed by tyrosinase. All the Gly-peptides and pseudostellarin A efficiently inhibited the oxidation of DOPA to dopaquinone (about 30%) while having low activity towards the hydroxylation of tyrosine. In particular, the **AOG9** peptide showed the lower inhibitory activity towards *L*-Tyr hydroxylation, while at 2 µM concentration, it owned an inhibitory activity towards DOPA oxidation comparable to that of the other peptides. The **AG9** peptide showed a similar activity, comparable to that of **PS-A,** towards the two reactions.

Surprisingly, **AG6** and **PS-A** showed an unusual inhibitory activity towards tyrosinase; indeed, their inhibitory activity was lower when the concentration was higher (Figure 4). This unusual behavior will need to be the subject of further research. A possible explanation is the presence of additional low-affinity peptide-enzyme binding sites that may distrain part of the available binder peptide, but since the affinity for such additional sites is low, these interactions occur only at high peptide concentrations.

### 2.3. Docking Studies

**Figure 5:**
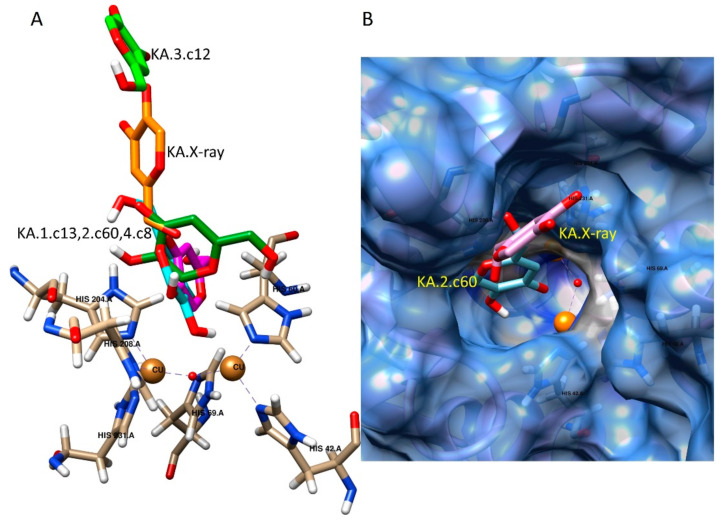
Representation of the catalytic site of *B. megaterium* tyrosinase in presence of kojic acid (KA) (PDB file 3NQ1). (**A**) Copper ions (orange balls), kojic acid in X-ray (orange), the best docking poses of kojic acid (magenta, green and pink), the six hystidines (turquois) coordinated to the copper ions. The red sphere represents a molecule of water. (**B**) The entrance of the catalytic site. KA in the X-ray structure (orange) and the best scoring position (violet). Copper ions (orange balls) and molecule of water (red ball).

In order to elucidate the interactions occurring among the tyrosinase catalytic site and the investigated cyclopeptides, molecular docking studies were carried out using the structure of the tyrosinase isolated from *Bacillus megaterium* crystallized in the presence or not of a molecule of kojic acid (PDB files: 3NQ1 and 3NM8, respectively) [26]. Two Cu(II) ions bridged with one molecule of water and surrounded by six histidine residues are present in the active site of the enzyme (Figure 5).

Each copper ion coordinates with three histidines identified as His42, His60, His69 for one ion and His204, His208, His231 for the second one. Recently, it has been supposed that both monophenols and *o*-diphenols bind to the same copper ion since a reorganization of the phenolate around the two copper ions occurs in the diphenolase form. The presence of one molecule of water bridged with Cu(II) and the absence of bonds among dioxygen and Cu(II) is representative of the *met* form of the protein involved in the diphenolase activity [27]. The crystal structure resolved with an inhibitor as kojic acid (KA) showed that this molecule is positioned at the entrance of the catalytic site, at 7 Å away from the copper center. The acid activates strong interactions with amino acid residues Phe197, Pro201, Asn205, and Arg209 that characterize the rim of a channel facing the catalytic site and stabilize the entrance of KA (Figure 5B) [26]. In this representation, KA does not activate strong interactions with the molecule of water bonded to Cu(II) because the acid is positioned in proximity to the entrance of the active site. On the contrary, the best pose of molecular docking of KA (roughly 73%) evidenced strong interactions (hydrophobic and H-bond) with the His204, Asn205, and His208 amino acids positioned in the catalytic site (Tables S1 and S2 of Supplementary Materials).

The mechanism of interaction with the active site of the enzyme is similar for all the analyzed cyclopeptides. Due to the relatively high dimensions and features of the molecules, they are positioned on the active site as a cap looked at by interactions such as hydrogen bonding and hydrophobic interactions with some side chains of the cyclopeptide oriented in the directions of the two copper atoms of the active site. Although antamanide and its analogs can form complexes with different metal ions, there is anyhow no evidence of the formation of stable complexes with the Cu(II) present in the catalytic site of enzymes. The conformation of the two best dockings poses of antamantides is pretty similar (Figure S3), evidencing a symmetry in the molecule with a K*i* that ranges from 2.59 to 3.27 μM. During the catalytic process that involves modulation of the molecular volume of the catalytic site exerted by Arg209, the antamanide structure is likely less flexible than its derivatives, allowing the entrance of the substrate even at a high concentration of the compound.

**Figure 6:**
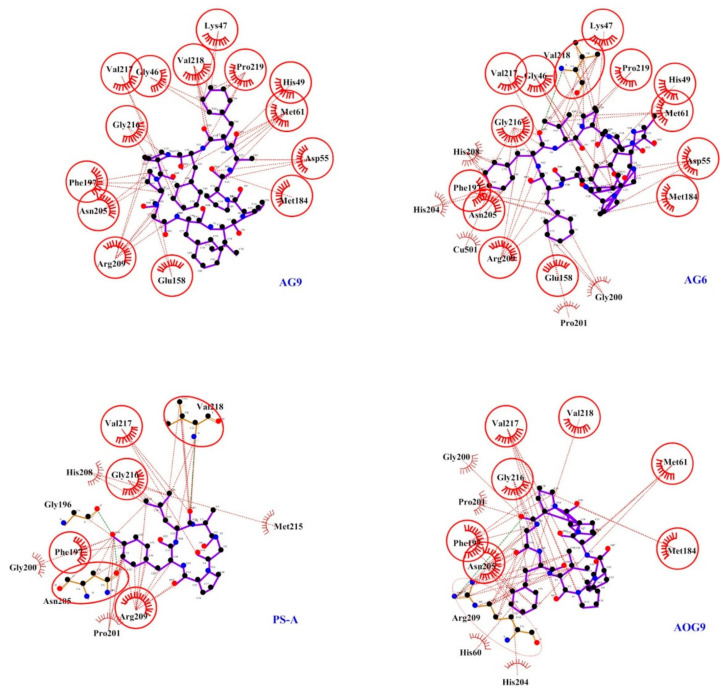
Hydrophobic interactions of the lowest docking poses of **AG9**, **AG6**, **PS-A** and **AOG9** with the catalytic site of *B. megaterium* tyrosinase (PDB file: 3NM8) performed with LigPlot+ [30].

All cyclopeptides interact with a set of amino acid residues Met61, Phe197, Pro201, Asn205, Arg209, Gly216, Val217, and Val218 positioned at the entrance of the channel facing the catalytic center with an estimated K*i* ranging µM values (Table S1). It has been suggested that Val218 plays a key role in the catalytic process. Likely, the amino acid, conformationally flexible and close to the first copper ion, is involved in the catalytic process, controlling the entrance of the substrate into the active site [28]. The different interactions that Val218 activates with each cyclopeptide can compromise the main function of the amino acid in directing the substrate correctly. Analogously, Arg209 positioned in proximity to the entrance of the catalytic site can expand or contract the molecular volume of the active site by virtue of the high conformational flexibility of this amino acid that allows modulating the interactions with the surrounding amino acids residues [28,29]. As shown for KA, the set of amino acids Phe197, Pro201, Asn205, and Arg209 appears to stabilize the cyclopeptides positioned at the rim of the site; in this conformation, the cyclopeptide would disturb the entrance of the ligand. The affinity that each cyclopeptide exerts on the amino acids surrounding this part of the site would affect the role of Arg209 and Val218 in the enzyme activity. Indeed, only **AG9** and **PS-A** bind to Val218, Asn205, and Arg209 with strong hydrophobic and H-bonds interactions, whereas **AG6** activates strong hydrophobic interactions only with Val218 and Arg209, confirming the less inhibitory activity observed in the spectrophotometric assay (Figure 6, Tables S1 and S2). **AG6** and **AG9** are antamanide-derivatives that differ from each other by the position of a glycine that substitutes phenylalanine in the parent molecule (Table 1). Molecular docking of **AG6** and **AG9** evidenced that the presence and the position of a glycine residue in **AG6** and in **AG9** modify the conformation of the cyclopeptide, influencing its ability to activate interactions with the amino acids in the proximity of the catalytic center. Although the best docking pose of antamanide establishes a strong binding affinity between phenyl moieties of the molecule with Asn205 and Arg209 of the protein, the cyclopeptide activates only one hydrogen bond with the oxygen of the peptide bond of proline 7 with Val218, confirming the crucial role that this amino acid plays in the catalytic process of tyrosinase [28]. The cyclopeptide assumes a conformation that permits the entrance of the substrate and, in contrast with the other cyclopeptides, no interaction with any histidine residue of the catalytic center was estimated for antamanide (Figures S2 and S3 in Supplementary Materials). No inhibitory activity was detected for antamanide by spectrophotometric tyrosine assays.

In **AG9**, two strong hydrophobic interactions (π-π stacking) were evidenced: proline 7 and glycine 9 of the cyclopeptide with Arg209 and proline 8 with Asn205. Leucine 4 of **PS-A** strongly interacts with the aliphatic chain and the carbonyl of Val218, whereas cross-bridge interactions with the OH-phenolic group of tyrosine 3 and Asn205 were calculated. **AOG9**, the octapeptide lacking Phe5 and Phe6 residues compared to **AG9,** possesses a conformation that generates 99% of docking poses at the lowest estimated free energy of binding of −7.55 Kcal/mol.

**Figure 7:**
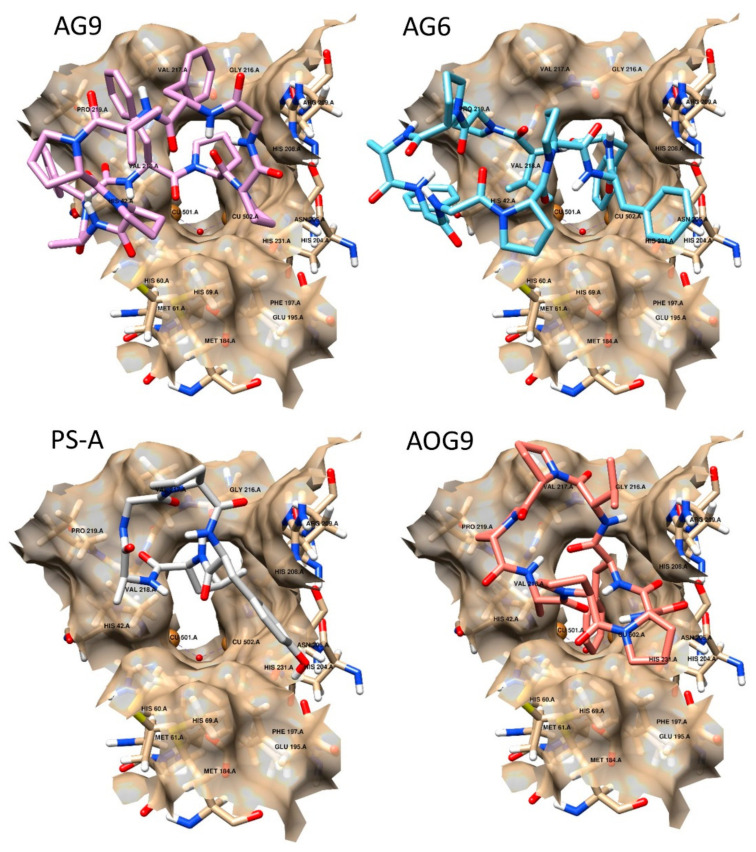
Docking simulation of cyclopeptides with *B. megaterium* tyrosinase protein (PDB file: 3NM8) evidencing the representative amino acids residues of the catalytic site, **AG6** (light blue), **AG9** (pink sticks), **AOG9** (light red sticks) and **PS-A** (grey sticks).

The molecular conformation of **AOG9** forces the molecule to assume only one energetically favored position at the entrance of the catalytic site in such a way to hamper any strong hydrophobic interactions with Val280 as **AG9**, **AG6** and **PS-A** activate in all own lower conformations of docking poses. This assumption is in agreement with the spectrophotometric assay that confirms a weak tyrosinase inhibition occurred in the presence of **AOG9**. Figure 7 represents the lowest docking poses of **AG9**, **AG6**, **PS-A,** and **AOG9** inside the catalytic site of the protein.

Cyclopeptides **AG6**, **AG9,** and, to a lesser extent, **PS-A** interact with a set of amino acids located in the rim of the channel and are representative of a wide area of the protein: Gly46, Lys47, His49, Asp55, Asn57.

While the best docking poses of **AG6**, **AOG9** and **PS-A** interact with almost one histidine of the catalytic center, supposing a possible inhibition, no interaction with histidine residues of the catalytic center has been predicted with the best pose of **AG9**. The conformational flexibility of **AG9** permits the molecule to cover the entrance of the catalytic site thanks to strong interactions with Gly46, Lys47, Asp55, Asn57, Met61, and Glu158, leading to suppose that the real role of these cyclopeptides is to hamper the entrance of the substrate. In particular, interactions with Glu158 and Pro201, positioned at the opposite side with respect to the set of amino acids Gly46, Lys47, Asp55, Asn57, and Met 61, allow **AG9** to cover the whole perimeter of the rim of the channel (Figure S4 in Supplementary Materials).

## 3. Materials and Methods

### 3.1. Peptide Synthesis

Fmoc-protected amino acids, preload Wang resin, and coupling reagents (HBTU and HOBt) were purchased from Iris Biotech (Marktredwitz, Germany). DMF, DIEA, and solvents were obtained from Sigma-Aldrich (Milan, Italy).

Peptides were synthesized by manual solid phase using Fmoc chemistry on 0.06 mmol scale. HBTU/HOBt activation employed a three-fold molar excess (0.24 mmol) of Fmoc-amino acids in DMF for each coupling cycle. Coupling times were 40 min. Fmoc deprotection was performed with 20% piperidine in DMF. Coupling yields were monitored on aliquots of peptide-resin either by Kaiser test for the amino groups or by evaluation of Fmoc displacement [31]. The cleavage from the resin was performed by treatment with TFA-anisole-triisopropylsilane-H_2_O (95:2.5:2.0:0.5 *v*/*v*) (45 min). Peptides were cyclized in 1 mM DMF solution by addition of 2 eq. of DPPA in presence of solid Na_2_HPO_4_ (5 eq) [32].

Peptides were purified by preparative Reversed-Phase HPLC using a Shimadzu LC-8 (Shimazdu, Kyoto, Japan) system with a Vydac 218TP1022, 10 µ, 250 × 22 mm column (Dionex, Sunnyvale, CA, USA). The column was perfused at a flow rate of 12 mL/min with a mobile phase composed of solvent A (0.05% TFA in water) and solvent B (0.05% TFA in acetonitrile/water, 9:1 by vol.), and a linear binary gradient was used. The fractions containing the desired products were collected and lyophilized to constant weight.

Analytical HPLC analyses were performed on a Shimadzu LC-10 instrument fitted with a Jupiter C18, 10 µ, 250 × 4.6 mm column (Phenomenex, Torrance, CA, USA) using the above solvent system (solvents A and B), flow rate of 1 mL/min, detection at 216 nm. All peptides showed less than 1% of impurities.

Molecular weights of compounds were determined by ESI-MS on a Mariner (PerSeptive Biosystems, Framingham, MA, USA) mass spectrometer. The mass was assigned using a mixture of neurotensin, angiotensin, and bradykinin at a concentration of 1 pmol/μL as external standard.

### 3.2. Tyrosinase Inhibition

The capability of cyclic peptides to inhibit the oxidation of Tyr by the tyrosinase/O_2_ oxidizing system was monitored by means of a UV-Vis spectroscopy method, carried out in a Shimadzu UV-Visible UV-2501 spectral spectrophotometer using Helma (Mülheim, Germany) duple chambers UV-cell (2 × 4.375 mm). Briefly, 880 µL of a Tyr solution (2 mM) and 900 µL of a mushroom tyrosinase solution (2000 U/mL, Sigma-Aldrich), both dissolved in 50 mM phosphate buffer (pH 6.8), were separately introduced in the duple UV-cell chambers. A total of 20 µL of an appropriate DMSO stock solution of inhibitors (1.8, 0.18, or 0.018 mM) were added to the chamber containing the Tyr solution. The tyrosinase activity in absence of inhibitors was detected by adding 20 µL of DMSO. A background UV-Vis spectrum was acquired in the 250–800 nm wavelength range with a 2.0 nm slit and fast speed before starting the reaction. Thereafter, the solutions were mixed, and UV-Vis spectra were acquired at different time points after mixing. Three replicates were performed for each experiment. Data processing was performed with UV Probe (Shimazdu, Kyoto, Japan) and OriginPro 2019 (v. 9.60) software (OriginLab Corporation, Northampton, MA, USA).

Purchased mushroom tyrosinase was used without further purification. Both L-tyrosine and tyrosinase solutions were filtered through a 0.45 μm syringe filter, aliquoted in Eppendorf tubes, and stored in freezer. The concentration of the two solution was determined spectrophotometrically (ε_Tyr_ at 274.6 nm = 1.420; ε_tyrosinase_ at 280 nm = 1.426) [33,34].

Tyrosinase-dependent diphenol and *o*-quinone formation was assessed as the appearance of a characteristic absorption band at about 304 and 477 nm, respectively. The inhibition activity was determined according to the following equation [33,34]:

% inhibition = 1 − [(D − C)/(B − A)] ∗ 100

where C and D are the absorbance values at either 304 or 477 nm in presence of inhibitor before mixing and upon reaching the maximum absorbance, respectively, while A and B are the corresponding values in absence of inhibitor.

### 3.3. Computational Methods

*Preparation ligand.* Model compounds were constructed with standard bond lengths and angles from the fragment database with MacroModel 5.5 (Schrödinger, LLC, New York, NY, USA, 2012) using a Silicon Graphics O2 workstation running on IRIX 6.3 Sybyl 6.2 (2001) (Sgi™, Mountain View, CA, USA). Minimization of structures conformational search was performed with the MacroModel/BachMin 6.0 program using the AMBER force field. An extensive conformational search was carried out using the Monte Carlo/energy minimization [35] (E_i_ − E_min_ < 5 kcal/mol, energy difference between the generated conformation and the current minimum). The atomic charges were assigned using the Gasteiger–Marsili method [36]. Representative minimum energy conformations of each compound were optimized using the Density Functional Theory (DFT), quantum chemistry program Gaussian 09 W [37] with method B3LYP/6-31G basis set [38]. Virtual analysis was performed, taking advantage of GaussView version 5.0 [39]. For the graphical display CHIMERA version 1.8 was used [40]. All ligands were docked with all bonds free to rotate.

*Structure refinement and computational docking procedures*. Binding of the compounds was analyzed using AutoDockTools 1.5.7rc1 and AutoDock4.2.5.1 docking programs [41]. The starting protein tyrosinase was prepared from the 2.00 Å resolution crystal structure deposited by Sendovski et al. [26] (PDB files: 3NM8). The binding site of 3NM8 was determined by comparing the position of the inhibitor (Kojic Acid) of the tyrosinase, as present in the 3NQ1 X-ray [26]. Provided the type of protein-containing inhibitor KA (PDB 3NQ1)**,** the docking was performed using a grid of 60 × 60 × 60 points, 0.375 Å spacing, and center −10, 20, −5, in order to circumscribe the interaction area of the catalytic site and therefore improving computation time. Then, considering that crystal structures 3NQ1 and 3NM8 are overlapping, the docking of the compounds was performed using crystal protein 3NM8 lacking in the ligand.

Additionally, some tests were run considering the whole enzyme to search other interesting sites. The crystallographic water molecules and Zn, Cl ion not involved in the catalytic process, were stripped. Conversely, the two copper ions and the molecule of water present in the catalytic site were conserved.

Hydrogen atoms were added using the ADT module of MGLTools 1.5.7rc1 suite program. The Gasteiger [36] charges of Autodock for the ligands and protein were used. The structures were docked using the Lamarckian genetic algorithm (LGA), utilizing the default grid spacing, treating the active docking site as a rigid molecule and the ligands as flexible, i.e., all non-ring torsions were considered active. The Lamarckian genetic algorithm (LGA) of up to 100 runs was employed with the settings of population size of 150 individuals, maximum number of generations, and energy evaluations of 27,000 and 25,000,000, respectively. Estimated inhibition constant (Ki_ex_) is modeled by the equation:

K*i*_ex_ = exp [(ΔG_ex_ ∗ 1000)/(R ∗ T)]

where ΔG_ex_ is a semiempirical free energy approximation (derived from molecular mechanics and experimental parameters), R is 1.98719 cal·K^−1^·mol^−1^, and T is 298.15 K. Notewothly, the result is not real free energy but only an approximation used in order to obtain an estimation of K*i* and to simplify the results for the comparison between experiment and docking procedures.

## 4. Conclusions

The identification of inhibitors of tyrosinase activity represents an attractive goal not only in the food industry, to preserve fruits and vegetables during storage, but also in the cosmetic and medicinal industries.

Here we investigated the capability of the natural bioactive peptide antamanide, isolated from mushrooms, and that of its analogs to inhibit the tyrosinase activity. While antamanide was not able to inhibit the tyrosinase activity, three glycine derivatives, **AG9**, **AG6**, and **AOG9,** were identified as tyrosinase inhibitors by spectrophotometric assays and computational studies. **AG9** showed higher inhibitory activity in comparison to **PS-A**, a natural and known tyrosinase inhibitor. Molecular docking performed on *B. megaterium* tyrosinase protein evidenced the binding affinity that cyclopeptides **AG9**, **AG6** and **AOG9,** and **PS-A** exert on the catalytic site of the protein, in agreement with the catalytic process suggested in previous studies for conventional tyrosinase inhibitors. The structural features of cyclopeptides **AG9**, **AG6,** and **AOG9** lead to suppose an additional behavior of the cyclopeptides at the entrance of the catalytic site that hampers the entrance of the substrate.

## Figures and Tables

**Table 1:** Sequence of antamanide analogues and pseudostellarin A (**PS-A**) peptide.

*Peptide*	*Sequence*									
	1	2	3	4	5	6	7	8	9	10
***Antamanide***	V	P	P	A	F	F	P	P	F	F
***AG6***	V	P	P	A	F	**G**	P	P	F	F
***AG9***	V	P	P	A	F	F	P	P	**G**	F
***AOG9***	V	P	P	A	**-**	**-**	P	P	**G**	F
***PS-A***	G	P	Y	L	A					

## Data Availability

Not applicable.

## Supplementary Materials

The following supporting information can be downloaded at: https://www.mdpi.com/article/10.3390/ijms23116240/s1. Reference [30] is cited in the Supplementary Materials.
