# Supplementary material for: Antamanide Analogs as Potential Inhibitors of Tyrosinase
Source: Int J Mol Sci. 2022 Jun 2;23(11):6240. doi: 10.3390/ijms23116240 (PMC9181589; doi:10.3390/ijms23116240)
Supplement: Supplementary file 1 [file ijms-23-06240-s001.zip › ijms-1714838-supplementary.pdf]

Antamanide Analogs as Potential Inhibitors of Tyrosinase

Claudia Honisch [1], Matteo Gazziero [1], Roberto Dallocchio [2], Alessandro Dessì [2], Davide Fabbri [2], Maria Antonietta Dettori [2], Giovanna Delogu [2] and Paolo Ruzza [1,*]

[1] Institute of Biomolecular Chemistry of CNR, Padova Unit, 35131 Padova, Italy; c.honisch@icb.cnr.it (C.H.); matteo.gazziero@gmail.com (M.G.)
[2] Institute of Biomolecular Chemistry of CNR, Sassari Unit, 07100 Sassari, Italy; roberto.dallocchio@icb.cnr.it (R.D.); alessandro.dessi@cnr.it (A.D.); davidegaetano.fabbri@cnr.it (D.F.); mariaantonietta.dettori@cnr.it (M.A.D.); giovanna.delogu@icb.cnr (G.D.)
* Correspondence: paolo.ruzza@cnr.it; Tel.: +39-049-827-5282

Supplementary Material

Table S1. Docking list of cyclopeptides with amino acids of catalytic site of *B. megaterium* tyrosinase protein (3NM8).

	Tested ligands	%	M.B.E.[a]	E.F.E.B.[b]	E.I.C., Ki[c]	Interactions, **HBond**
**1**	Antamanide.1.c36.r16	36	-7.05	-7.62	2.59 uM	Asp55 Met61 Met184 Phe197 Asn199 Gly200 Pro201 Asn205 **Arg209** Gly216 Val217 **Val218**
	Antamanide.2.c21.r10	21	-6.82	-7.48	3.27 uM	Met61 Met184 Phae197 Asn199 Gly200 Asn205 **Arg209** Gly216 Val217
	Antamanide.5.c14.r44	14	-6.70	-6.84	9.64 uM	Met61 Lys157 **Glu158** Arg209 Gly212 Gly213 Gly216 Val217 **Val218** Pro219
**2**	AG6.1.c20.r33	20	-6.80	-7.92	1.57 uM	Gly46 Lys47 His49 Asp55 Met61 Met184 Phe197 Gly200 Pro201 His204 Asn205 His208 Arg209 Gly216 Val217 **Val218** Pro219
	AG6.4.c30.r66	30	-6.45	-7.04	6.89 uM	Met61 Met184 Phe197 Gly200 Pro201 Asn205 His208 Arg209 Gly216 Val217 Val218
**3**	AG9.3.c28.r85	28	-5.29	-6.52	16.71 uM	Gly46 Lys47 Asp55 Met61 Met184 Phe197 Asn205 Arg209 Gly216 Val217 Val218 Pro219
	AG9.5.c15.r27	15	-5.62	-6.19	29.00 uM	Gly46 His49 Asp55 Asn57 His60 Met61 Phe197 Asn205 Arg209 Gly216 Val217 **Val218** Pro219
	AG9.12.c33.r20	33	-4.89	-4.92	245.95 uM	Met61 Glu158 Met184 Phe197 Pro201 **Arg209** Gly216 Val217 Val218 Pro219
**4**	AOG9.1.c99.r28	99	-7.31	-7.55	2.90 uM	His60 Met61 Phe197 Pro201 His204 Asn205 **Arg209** Gly216 Val217 Val218 Pro219
**5**	PS-A.2.c10.r49	10	-5.89	-5.94	44.51 uM	**Gly196** Phe197 Gly200 Pro201 **Asn205** His208 **Arg209** Gly216 Val217 **Val218**
	PS-A.5.c22.r7	22	-5.80	-5.89	48.12 uM	His60 Met184 Phe197 Gly200 Pro201 His204 Asn205 His208 **Arg209** Gly216 Val217 Val218
	PS-A.6.c24.r15	24	-5.73	-5.76	59.86 uM	Gly46 Asn57 His60 Met61 **Glu158** Phe197 Pro201 **Arg209** Gly216 Val217 **Val218**
**6**	KojicAcid.1.c13.r51	13	-4.22	-4.23	789.57 uM	His60 **Glu195** His204 **Asn205** His208 **Gly216** Val218
	KojicAcid.2.c60.r16	60	-3.70	-4.00	1.17 mM	His204 **Asn205** His208 **Arg209** Gly216
	KojicAcid.3.c12.r100	12	-3.51	-3.56	2.47 mM	**Glu158 Phe197 Gly200** Pro201 **Arg209**
	KojicAcid.4.c8.r91	8	-3.49	-3.50	2.72 mM	**Asn205** His208 **Arg209** Gly216 Val217 **Val218**

[a] M.B.E.: Mean Binding Energy,

[b] E.F.E.B.: Estimated Free Energy of Binding,

[c] E.I.C., Ki: Estimated Inhibition Constant, Ki.

Table S2. H-bonds list of cyclopetides with amino acids of catalytic site of *B. megaterium* Tyrosinase protein (3NM8)

Hydrogen bond interactions

pose	Tested Ligands	%	H-bond	Ligand Atom	Protein Atom	Distance (Å) [c]	Ang. (°)[c]
1	Antamanide	36	2	O58(OA) [a]	Arg209:1HH1(HD)	2.294	151.22
				O39(OA)	Val218:HN(HD)	2.238	157.69
2		21	1	O29(OA)	Arg209:1HH1(HD)	2.261	143.86
5		14	2	O39(OA)	Glu158:HN(HD)	1.905	136.71
				O9(OA)	Val218:HN(HD)	2.072	145.82
1	AG6	20	1	O58(OA)	Val218:HN(HD)	2.557	114.15
4		30	0	---------------	---------------	------------	----------
3	AG9	28	0	---------------	---------------	------------	----------
5		15	1	O29(OA)	Val218:HN(HD)	1.832	142.89
12		33	1	O46(OA)	Arg209:HE(HD)	2.181	161.25
1	AOG9	99	2	O37(OA)	Arg209:1HH1(HD)	**2.091**	**125.99**
				O43(OA)	Arg209:2HH1(HD)	**2.470**	**174.94**
2	PS-A	10	4	H37(HD) [b]	Gly196:O(OA)	2.486	82.60
				H37(HD)	Asn205:OD1(OA)	2.093	140.87
				O15(OA)	Arg209:HE(HD)	2.487	83.07
				O27(OA)	Val218:HN(HD)	1.623	171.78
5		22	1	O10(OA)	Arg209:1HH1(HD)	2.006	118.38
6		24	3	H37(HD)	Glu158:OE2:(OA)	2.022	158.66
				O36(OA)	Arg209:1HH1(HD)	1.916	164.36
				O10(OA)	Val218:HN(HD)	1.979	172.48
1	Kojic Acid	13	3	H10(HD)	Glu195:OE1(OA)	2.000	162.49
				O9(OA)	Asn205:1HD2(HD)	1.855	147.85
				H12(HD)	Gly216:O(OA)	2.212	111.59
2		60	2	H10(HD)	Asn205:O(OA)	2.129	137.75
				O9(OA)	Arg209:2HH1(HD)	2.152	162.22
3		12	4	H10(HD)	Glu158:OE2:(OA)	2.075	111.96
				H12(HD)	Phe197:O(OA)	2.061	150.81
				O11(OA)	Gly200:HN(HD)	2.416	135.33
				O9(OA)	Arg209:1HH1(HD)	1.862	168.53
4		8	3	H12(HD)	Asn205:O(OA)	1.770	136.66
				O7(OA)	Arg209:2HH1(HD)	2.116	162.37
				O9(OA)	Val218:HN(HD)	2.062	165.48

[a] Oxygen acceptor, [b] Hydrogen donor, [c] Cross-bridge H-bond interactions with the same aa are listed in bold.

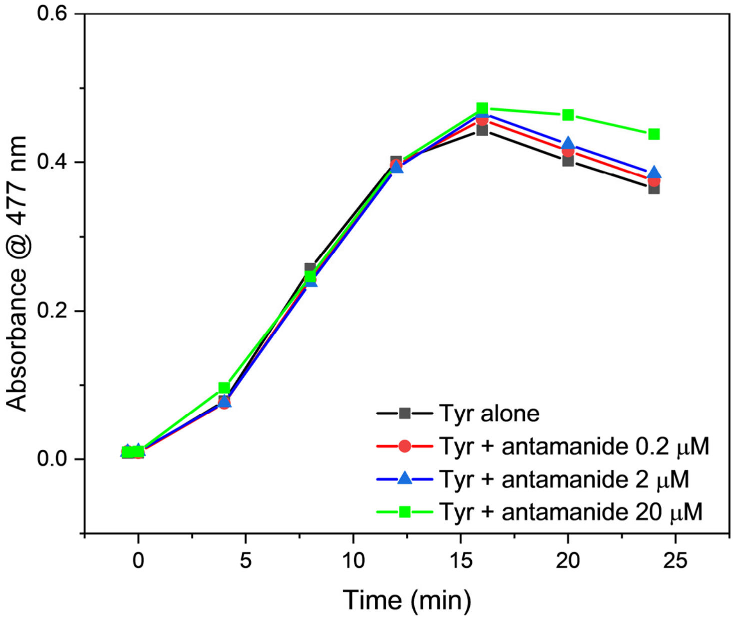

Figure S1. Influence of antamanide (at different concentrations, indicated) on the time-course of tyrosine oxidation by the tyrosinase/O_2 oxidizing system.

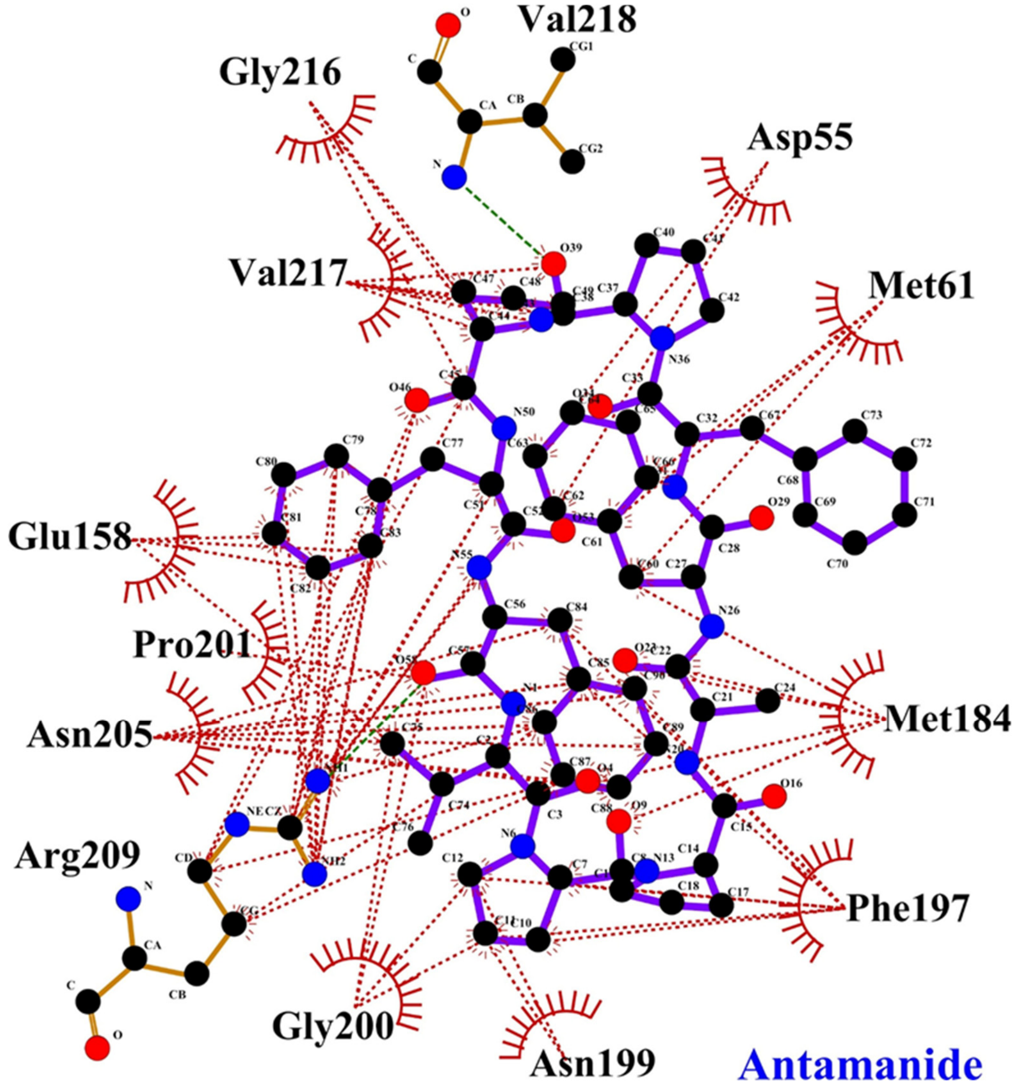

Figure S2. Hydrophobic interactions of the lowest docking pose of Antanamide with the catalytic site of *B. megaterium* tyrosinase protein (3NM8) and performed with LigPlot+ [Laskowski, R. A.; Swindells, M.B. LigPlot+: Multiple ligand-protein interaction diagrams for drug discovery. *J. Chem. Inf. Model.* **2011**, *51*, 2778–2786.].

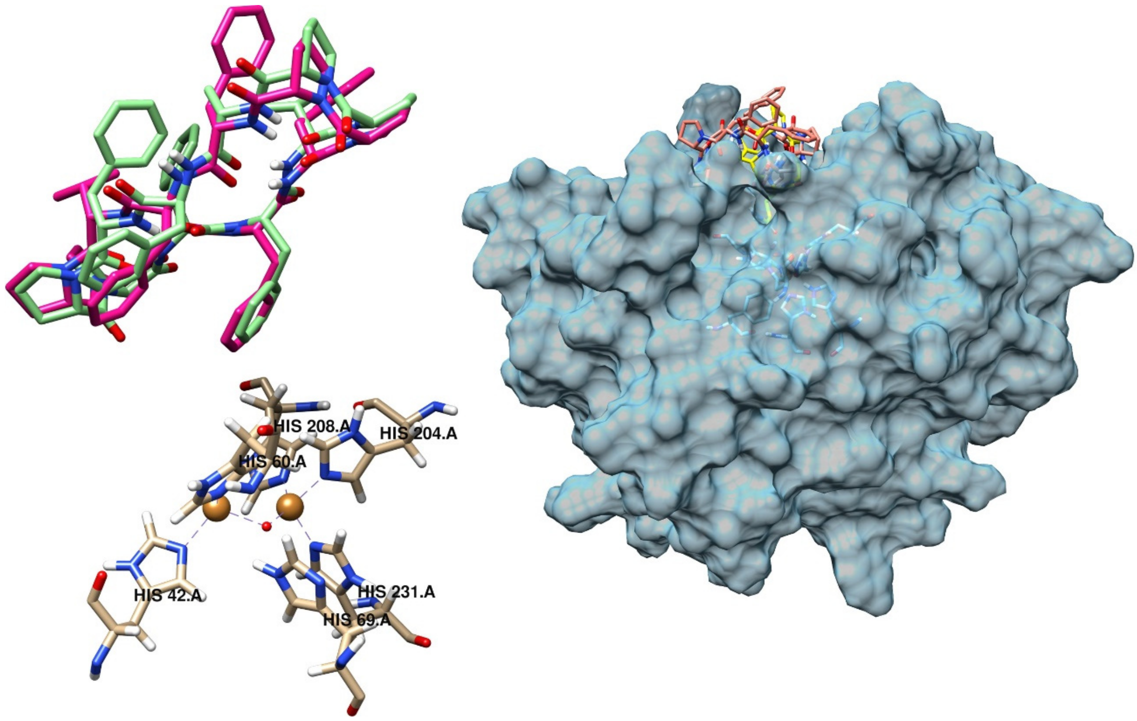

Figure S3. Interaction between amino acids residues and the two best poses of Antamanide (green and pink stick). Catalytic site of Tyrosinase enzyme and the two best poses of Antamanide (left) and in the presence of the whole projection of the protein (right).

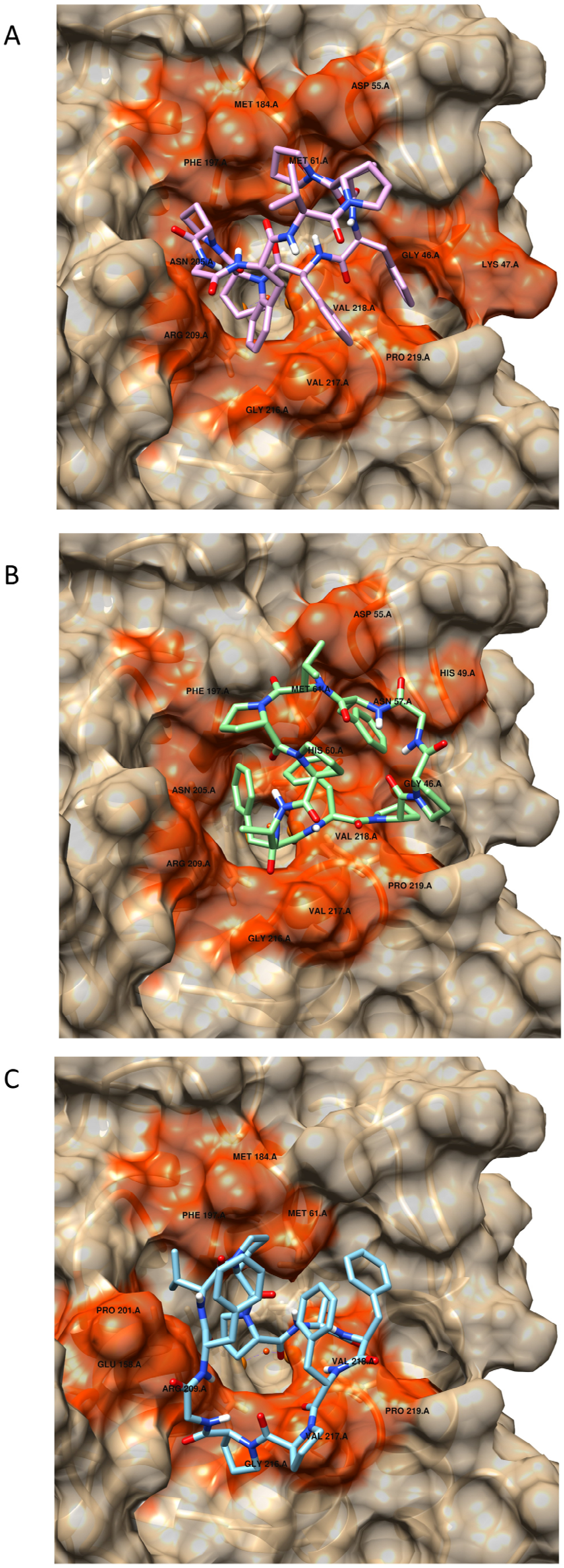

Figure S4. Interaction between amino acids residues at the entrance of the catalytic site of *B. megaterium* Tyrosinase and the different pose 3.c28 (**A**), 5.c15 (**B**) and 12.c33 (**C**) of **AG9**.
